# Imported West Nile Virus Infection in Europe

**DOI:** 10.3201/eid0906.02-0723

**Published:** 2003-06

**Authors:** P.E. Charles, H. Zeller, B. Bonnotte, A.L. Decasimacker, J.B. Bour, P. Chavanet, B. Lorcerie

**Affiliations:** * Dijon University Hospital, Dijon, France; † Institut Pasteur, Lyon, France

**To the Editor**: We report the case of an 82-year-old man, recently arrived in France from Atlanta, Georgia, USA, who had chills and fever in late August 2002. He left Atlanta on August 26, 2002, and spent a week in Paris before he reached Burgundy. On day 9, after his arrival in the town of Dijon, he had chills and fever (40°C), weakness, malaise, diarrhea, and headache. He was then admitted to Dijon University Hospital. Physical examination indicated hyporeflexia, mild changes in mental status, and no neck stiffness. No lumbar puncture was performed.

Laboratory findings included the following: hyponatremia, 129 mmol/L (normal range 135–145 mmol/L); C-reactive protein, 13 mg/L (normal <3 mg/L); lymphocyte count, 500 cells/mm^3^ (normal range, 1,000–4,000 cells/mm^3^); positive antinuclear antibodies, 1/160 (homogenous); and positive anti-DNA antibodies, 76 Word Health Organization (WHO) U (normal <39 WHO U). Blood and urine samples remained sterile. Results of the chest roentgenogram and electrocardiogram were normal.

Serum immunoglobulin (Ig) M antibodies to West Nile virus (WNV) were detected by using antibody-capture enzyme-linked immunosorbent assay (ELISA); IgG antibodies were not found by ELISA. A second serologic test performed 2 weeks later in the United States also showed specific IgM antibodies to WNV. The diagnosis of WNV infection was thus established. Four days after admission, the patient no longer had a fever, and his mental status was normal.

WNV infection is a potentially lethal mosquito-borne infection (*1*). Since 1994, many notable outbreaks have occurred (*2*–*4*). The virus emerged in New York, New York, USA, in 1999, and WNV infection is likely to become enzootic in the United States (*5*). In 2002, WNV was reported in 43 states, and human cases were reported in 33 states (*6*). The first human case in Georgia was described in 2001.

We report the first imported case of WNV infection in Europe, on the basis of criteria established by the Centers for Disease Control and Prevention. WNV infection was suspected because our patient arrived from an area where WNV is epidemic during the late summer. Clinical findings were similar to those described in previous cases (*5*). Encephalitis was suspected because the patient showed a reversible alteration of mental status and headache. Although hyponatremia and lymphocytopenia have previously been reported in cases of WNV infection, positive anti-DNA antibodies is a finding of particular interest. However, the mild elevation of antibody titers is common in other viral infections, especially in those caused by members of the *Flaviviridae* family such as hepatitis C virus (*7*). Although the prognosis of WNV infection is generally poor in elderly patients, our patient had a good outcome (*5*,*8*).

In conclusion, physicians in western Europe should be aware of the risk of WNV infection among travelers from a disease-endemic area such as the United States, especially during late summer. Specific antibody detection tests should be performed in such patients with unexplained fever, particularly when they show evidence of neurologic disease. Suspected and confirmed cases can then be quickly reported to health departments, leading to an improvement in the public health response. However, imported cases like this one are not likely to contribute to the spread of WNV infection in Europe. Indeed, human viremia levels seem too low and of insufficient duration to allow the infection of competent mosquito vectors and the subsequent transmission of the virus to other hosts, such as horses or humans (*9*).
